# Stochastic dynamics of virus capsid formation: direct versus hierarchical self-assembly

**DOI:** 10.1186/2046-1682-5-22

**Published:** 2012-12-17

**Authors:** Johanna E Baschek, Heinrich C R Klein, Ulrich S Schwarz

**Affiliations:** 1 Institute for Theoretical Physics, University of Heidelberg, Heidelberg, Germany; 2 BioQuant, University of Heidelberg, Heidelberg, Germany

## Abstract

**Background:**

In order to replicate within their cellular host, many viruses have developed self-assembly strategies for their capsids which are sufficiently robust as to be reconstituted *in vitro*. Mathematical models for virus self-assembly usually assume that the bonds leading to cluster formation have constant reactivity over the time course of assembly (*direct assembly*). In some cases, however, binding sites between the capsomers have been reported to be activated during the self-assembly process (*hierarchical assembly*).

**Results:**

In order to study possible advantages of such hierarchical schemes for icosahedral virus capsid assembly, we use Brownian dynamics simulations of a patchy particle model that allows us to switch binding sites on and off during assembly. For T1 viruses, we implement a hierarchical assembly scheme where inter-capsomer bonds become active only if a complete pentamer has been assembled. We find direct assembly to be favorable for reversible bonds allowing for repeated structural reorganizations, while hierarchical assembly is favorable for strong bonds with small dissociation rate, as this situation is less prone to kinetic trapping. However, at the same time it is more vulnerable to monomer starvation during the final phase. Increasing the number of initial monomers does have only a weak effect on these general features. The differences between the two assembly schemes become more pronounced for more complex virus geometries, as shown here for T3 viruses, which assemble through homogeneous pentamers and heterogeneous hexamers in the hierarchical scheme. In order to complement the simulations for this more complicated case, we introduce a master equation approach that agrees well with the simulation results.

**Conclusions:**

Our analysis shows for which molecular parameters hierarchical assembly schemes can outperform direct ones and suggests that viruses with high bond stability might prefer hierarchical assembly schemes. These insights increase our physical understanding of an essential biological process, with many interesting potential applications in medicine and materials science.

## Background

The structure and dynamics of viruses are a fascinating research subject not only from a biological, but also from a physical perspective [1]. In particular, they are a very instructive model system to study self-assembly of large protein complexes with a relatively clear biological function. As viruses do not show metabolic activity of their own, they need to infect host organisms in order to replicate. One key step during the replication process is the formation of the protein shell containing the viral genome. For many viruses, the capsid formation is sufficiently autonomous that it occurs even *in vitro*[2]. This robustness of the process guarantees successful replication within the dynamic and heterogeneous environment of a living cell. Although virus shell formation is considered as a paradigm for the self-assembly of protein complexes [3], its underlying principles are far from being fully understood. Progress in our understanding of virus assembly would increase our knowledge of a process of large biological and medical relevance as well as help to advance new self-assembly strategies in materials science applications [4].

A large variety of mathematical models and simulation approaches has been developed to gain insight into the dynamics of capsid formation from a theoretical perspective. In these approaches the characteristics of protein association and dissociation processes were analyzed depending on parameters like interaction strength, subunit geometry or temperature. The employed techniques range from large-scale Molecular Dynamics (MD) simulations with only a modest amount of coarse-graining of the atomic details [5] through various schemes of coarse-grained MD [6-11] to patchy particle simulations with interaction potentials [12,13]. A thermodynamic framework for assembly of icosahedral viruses has been established by Zlotnick and coworkers [14-20]. In general, these studies have revealed that the formation of complete virus capsids requires intermediate bond stability. If interaction strength is too high (or, equivalently, temperature too low), the system becomes kinetically trapped in intermediates which cannot rearrange anymore due to the strong binding. If interaction strength is too low (or, equivalently, temperature too high), the target structure is not sufficiently stable. Another mechanism which can prevent complete capsid formation is the occurrence of misfits, leading to structural polymorphism as often studied with MD-schemes allowing for cluster distortions [21-23].

Due to the large number of single building blocks assembling during virus formation (the simplest icosahedral capsid, T1, has already 60 protein components), there is a multitude of topologically possible assembly pathways. Similar to protein folding, the dominance of few key structures is believed to limit the number of pathways and to speed up the process [14]. In this respect it has been observed that some viruses have developed mechanisms to orchestrate self-assembly by regulating the reactivity of their binding sites [24,25]. This switching establishes a hierarchy in the formation of transient intermediates during the assembly process. In a number of experiments, partly supported by theoretical calculations, it has been shown that intermediates of pentameric and hexameric symmetry are of special importance for the assembly process of icosahedral viruses [3,26-32]. Early observations of *in vitro* assembly of phages and small viruses revealed pentamer sub-structures to play a key role [26,27]. Experiments on Brome Mosaic Virus [28], Cowpea Chlorotic Mottle Virus [30], Human Papillomavirus [31] and Simian Virus 40 *in vivo* and *in vitro*[32] explicitly report capsid assembly from pentameric capsomers. A model for the assembly of Cowpea Chlorotic Mottle Virus suggests that its protein shell assembles from pentamers as well as from trimers of dimers (hexamers) [3].

Despite the described variety of computational approaches used for virus assembly, to our knowledge the effect of a state-dependent activation of binding sites during the assembly process (*hierarchical assembly*) has not been explored yet from the theoretical point of view. Although some models consider assembly from pentameric and hexameric clusters, these subunits at the same time represent the smallest entities of the system and their formation from single proteins is not included [10,21,33]. Here we investigate the effect of a binding hierarchy on the assembly of icosahedral viruses by comparison of hierarchical and non-hierarchical (direct) assembly from single monomers. We use Brownian Dynamics simulations with reaction patches which have previously been used to study transport-limited protein reactions [34,35]. Our approach assumes well-defined capsid geometries (in the spirit of local rules) and does not require the use of interaction potentials. This makes our simulations relatively fast, but does not allow us to study structural polymorphism. One particular strength of our approach is that it implements the correct mobility matrix for each possible geometry of the assembling clusters [34]. Another advantage which is exploited here is that one can easily implement hierarchical assembly by an event-driven switching of patch reactivity.

This paper is organized as follows. We first give an overview of the simulation framework and the implemented geometries. Then we present our results for direct and hierarchical assembly of T1 virus capsids. The analysis of T1-assembly is completed with a comparison of the two assembly mechanisms and a discussion of the effect of an increased number of initial monomers. We then explain our results for direct and hierarchical assembly of the more complex T3 virus. They are followed by a detailed analysis of the formation of individual capsomers, which includes a master equation approach. The paper closes with concluding remarks and an outlook to potential future applications of our approach.

## Methods

### Outline of the computer simulations

**Figure 1:**
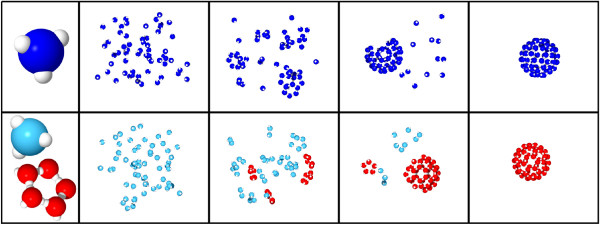
**Visualization of T1 Capsid Assembly.** The snapshots from the computer simulations show the course of T1 virus capsid formation for direct (top) and hierarchical assembly (bottom) from *n*_*f*_=60 single monomers. In hierarchical assembly, a color change of the proteins from blue to red indicates the switch of binding characteristics upon completed formation of a pentameric capsomer.

To study virus assembly we use a Brownian dynamics approach with patchy particles which has been developed before to investigate diffusion and association of model proteins and their complexes [34,35]. Single proteins are modeled as hard, spherical particles with equal radius. They are equipped with a specific number of reaction patches representing the binding sites. The geometry of the virus capsid is coded in the position of the reaction patches on the spheres. Assemblies of several proteins are treated as rigid objects whose diffusive characteristics are calculated on the fly upon formation [36]. In each simulation step the particles are propagated according to their translational and rotational diffusive properties, followed by possible association and dissociation steps. Binding of two proteins is implemented as a two-step process following the notion of the encounter complex [37]. Upon diffusional overlap of two reaction patches, binding occurs stochastically with a predefined patch-specific rate *k*_*a*_. Thus, the probability for the transition from encounter to a bound state within a given timestep *Δt* is *p*_bind_=*k*_*a*_*Δt*. If the bond formation is accepted, the binding partners instantaneously click into their predefined relative orientation, assuming that this processes is much faster and less stochastic than diffusion. The repositioning is distributed among the two clusters according to their diffusive weights. If this reorientation leads to a steric overlap of the two associating partners with each other or with other protein complexes, binding is rejected and the old positions and orientations of the clusters are used for the next simulation step. Similarly to association, dissociation of an existing bond occurs stochastically with the bond specific rate *k*_*d*_. Thus, a bond is disrupted within *Δt* with the probability *p*_break_=*k*_*d*_*Δt*. If the broken bond was the only connection between two clusters, both are propagated independently in the following simulation step. The simulation algorithm is combined with a visualization routine which enables us to follow the assembly process. Some representative snapshots of the step-wise assembly of a T1 virus capsid are shown in Figure 1. While the upper row shows direct assembly from 60 monomers (dark blue), in the lower row monomers (light blue) first have to form pentamers (red), which then in turn form the complete capsid (hierarchical assembly). Corresponding movies are provided as Additional files 1 and 2, respectively. 

### Capsid geometries

The capsid geometries follow the well-established Caspar-Klug scheme where the quasi-equivalent positions in the scaffold of an icosahedral capsid are represented by different types of monomers [38]. The structural complexity is described by the triangulation number T derived from the capsid geometry. T is restricted to certain integer values (T=1,3,4,7,9,...) and denotes the number of protein types which are needed to form a full icosahedral shell. The total number of monomers per capsid is *n*_*f*_=60 T. The icosahedron vertices represent points around which the proteins cluster into close-packed arrays. The proteins grouped around the twelve vertices (which represent axes of fivefold symmetry) form pentamers, while the triangular faces of the icosahedron are covered with hexamers. Every scaffold consists of 12 pentameric and 10·(*T*−1)hexameric capsomers. In our description we restrict the effect of growing complexity (T > 1) to the hexamers. Thus, every hexamer contains (T−1) different proteins, so that the number of hexameric subunits as well as the number of individual components of each hexamer increase with T. We want to point out that this scheme for dividing virus geometries into ringlike subunits represents only one out of several possible realizations. Following previous approaches to the characterization of icosahedral geometries [39], we use a set of local rules to define the bond angles between the individual particles. In this way, the resulting structure is encoded in the bond properties of the elementary subunits. Due to the high symmetry of the viral capsid, only a small number of different bonds is sufficient to define a unique target geometry. We note that the exact definition of bond properties impedes the formation of aberrant cluster structures. Therefore the approach used here does not allow us to study structural polymorphism.

### Direct and hierarchical assembly

Direct assembly is defined as the formation of a capsid from monomers whose bond properties remain unchanged throughout the whole simulation. Thus every reaction patch is active at all times. In contrast to this unconstrained assembly mechanism, hierarchical assembly is decomposed into multiple steps of switching of patch reactivity depending on the configuration of the particles. Since pentameric and hexameric rings have been identified as key subunits in the assembly process, we implement hierarchical assembly as switching of reactivity upon formation of these structures. Initially only the two patches leading to assembly of pentameric or hexameric ring structures are active on every single protein (intra-capsomer bonds). These patches are locked once the ring has closed, so that the formation of these subunits is irreversible. Simultaneously to the locking of intra-capsomer patches the binding sites which connect the pentamers and hexamers with each other (inter-capsomer bonds) are activated so that in a second step, formation of the capsid proceeds via association of the capsomer rings. This collective switching in binding properties should not be confused with the conformational switching of individual subunits which has been used before to study structural polymorphism [21-23].

### Simulation details

At the beginning of each simulation run the single proteins are placed at random, non-overlapping positions in a cubic periodic boundary box. From this configuration we let the system evolve according to the algorithm described above with a constant timestep *Δt*corresponding to a real time of 0.1 ns. A trajectory (one simulation run of predetermined finite length) is considered as successful if a complete virus shell is formed within the simulation time. The diffusive properties used here correspond to a temperature value of T=293 K and a viscosity value of *η*=2·10^−3^Pa s. The single proteins have a radius of *R*=1 nm and a patch radius of *r*=0.4 nm with the center of the spherical patches placed at the surface of the protein. We choose the same initial concentrations for all simulations of one virus geometry. To observe a considerable number of association events within a reasonable time we use relatively high concentrations of several mM. Although these concentration values exceed those applied in experimental setups (several *μ*M [37]), this is a common practice in simulation approaches [7,11,34,35]. During the simulations we record the number of clusters of size *n*, *ν*_*n*_ (1≤*n*≤*n*_*f*_=60 T), as well as the first passage times (FTPs) of intermediates of specific sizes. The probability that some monomer belongs to a cluster of size *n* is *p*(*n*)=(*ν*_*n*_*n*)/*n*_*f*_. The sum of these probabilities is normalized to one. The average cluster size is given by

(1) $$\bar{n}=\overset{n_{f}}{\underset{n=1}{\sum }}p(n)n=\overset{n_{f}}{\underset{n=1}{\sum }}\frac{\nu_{n}n}{n_{f}}n\;.$$

## Results

### Overview

In this section we investigate the dynamics of virus capsid formation for direct and hierarchical assembly and compare them in order to identify their generic differences. To characterize the assembly performance, the yield (i.e. the relative number of successful trajectories within a given simulation time) and the first passage times (FPTs) of selected intermediates are recorded for different model parameters. We systematically compare both assembly mechanisms in a parameter space ranging from *k*_*a*_=3.0 ns^−1^ to 9.0 ns^−1^ and from *k*_*d*_=1.5·10^−3^ ns^−1^ to 1.95·10^−2^ ns^−1^. For the simulations of assembly of T1 capsids we use an initial monomer concentration of *c*=4.5 mM (60 particles in a cubic box with side length *L*=28nm). Investigation of T3 is carried out at an initial concentration of *c*=1.7 mM (*n*_*f*_=180, *L*=55nm). To classify different assembly regimes we distinguish between three different phases: the early, intermediate and final phases which we define to be delineated by the emergence of cluster sizes 1/3 *n*_*f*_, 2/3 *n*_*f*_ and *n*_*f*_, respectively.

### T1 direct assembly

**Figure 2:**
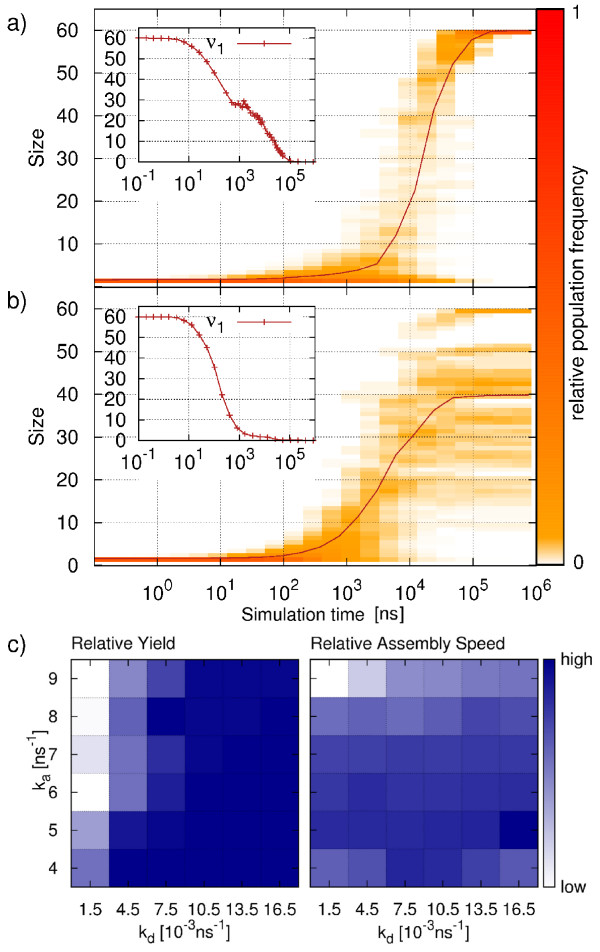
**T1 direct assembly. a)** and **b)** show the relative population of different cluster sizes as a function of time for a favorable (*k*_*a*_=5.0 ns^−1^, *k*_*d*_=13.5·10^−3^ ns^−1^) and a unfavorable (*k*_*a*_=8.0 ns^−1^, *k*_*d*_=1.5·10^−3^ ns^−1^) set of parameters, respectively. The average cluster size is shown as solid line. In the inset the monomer population *ν*_1_(*t*)is shown as a function of time. **c)** Parameter space analysis of direct assembly. Relative yield (left) and relative assembly speed (right) are depicted using a heat-map representation for various combinations of *k*_*a*_and *k*_*d*_. All data are obtained from 40 independent simulation runs.

Figures 2a and b show the temporal evolution of the relative population of all cluster sizes for one favorable and one unfavorable set of model parameters, respectively. The average cluster size $\bar{n}(t)$ (see Eq. 1) is shown as solid line and shows a sigmoidal shape. Starting from a full set of available monomers, we observe subsequent formation of dimers, trimers and then larger intermediate clusters. In the favorable case shown in Figure 2a, the distribution always stays close to the average and complete capsid formation is achieved. Remarkably, this successful case is also characterized by the relatively long persistence of a monomer pool (inset to Figure 2a). The persistence of a relatively high number of monomers during the intermediate assembly phase shows the system’s capability to reorganize and enables one dominant cluster to grow. In marked contrast, for the unfavorable case shown in Figure 2b, the distribution of intermediates considerably broadens. The average does not reach complete capsid formation, and the monomer pool is depleted much earlier. Here the intermediates are more restricted in undergoing recombinations, many trajectories become kinetically trapped and the average does not capture anymore the dynamics of the assembly process. In both cases, the assembly dynamics slow down during the final phase. This can, at least partly, be attributed to monomer starvation as the slow-down occurs when only very few monomers are left. The prominent features found here (sigmoidal kinetics, fast growth after lag time, kinetic trapping, monomer starvation in the final phase) have been found before also with coarse-grained MD-simulations [6,7,9]. 

The main difference between the two parameter sets used in Figure 2 is that the second (unfavorable) case leads to more stable intermediates (higher *k*_*a*_, lower *k*_*d*_). In Figure 2c we systematically investigate the effects of the bond parameters on direct assembly by comparing yield and assembly speed for different combinations of *k*_*a*_and *k*_*d*_. The upper left corner of the parameter plots represent strong bonds (high *k*_*a*_, low *k*_*d*_), while weak bonds are found in the lower right corner (low *k*_*a*_, high *k*_*d*_). The left plot shows the relative yield averaged over an ensemble of 40 trajectories. Direct assembly of T1 shows a large region of high yield for dissociation rate values above a threshold of around *k*_*d*_=10.5·10^−3^ ns^−1^. Below this value almost no successful assembly is observed. This is due to the limited possibilities of the intermediates to reorganize, which results in the occurrence of kinetically trapped structures. For low dissociation rates we also observe a dependency of the yield on the choice of *k*_*a*_. In this region, lowering of the bond breaking rate *k*_*d*_can at least partly be compensated by lowering of the association rate *k*_*a*_.

In the right plot of Figure 2c, we show the relative assembly speed as a function of model parameters. The assembly speed is defined as the inverse of the completion time of the capsid, *v* = 1/FPT(*n*_*f*_). Because this quantity can be obtained only for successful assemblies, here we average only over completed trajectories. In contrast to the relative yield, we see a clear dependence of the assembly speed on the association rate *k*_*a*_across the whole parameter space. Fastest assembly is observed for relatively low values of *k*_*a*_. The observation that relatively high association rates lead to slower assembly can be explained by the increasing tendency to form more than one large cluster in the early and intermediate phases. Thus, even for high dissociation rates, the necessary rearrangement of the clusters slows down the assembly process considerably. We also record a relatively high assembly speed at low *k*_*d*_values where only low yield is observed. Since the relative speed values are obtained by averaging over successful trajectories only, these results show that, if a full capsid is formed, it is completed within a short time.

To conclude, we see that the success of assembly in terms of yield is mostly determined by the choice of the dissociation rate *k*_*d*_. For low values of *k*_*d*_ the system becomes kinetically trapped, while large values of *k*_*d*_allow for the reorganization of the clusters. The relative assembly speed of successful trajectories is strongly influenced by the choice of *k*_*a*_. Here we identify an optimum at *k*_*a*_=5.0 ns^−1^, with speed being worse both at larger and smaller values. In agreement with previous studies, we observe that most efficient assembly (i.e. high yield combined with fast capsid completion) occurs at intermediate bond stability and that bond reversibility is an important requirement for successful capsid formation [9,40,41].

### T1 hierarchical assembly

**Figure 3:**
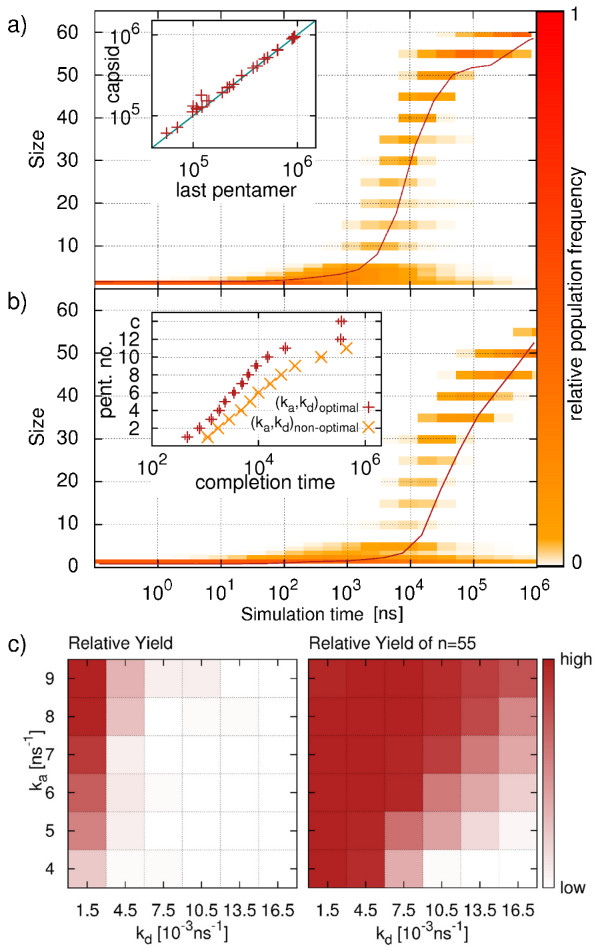
**T1 hierarchical assembly. a)** and **b)** show the relative population for a favorable (*k*_*a*_=8.0 ns^−1^, *k*_*d*_=1.5·10^−3^ ns^−1^) and unfavorable (*k*_*a*_=5.0 ns^−1^, *k*_*d*_=1.35·10^−2^ ns^−1^) set of parameters, respectively. The average cluster size is shown as solid line.In the inset of a) the FPT(*n*_*f*_) is plotted against the FPT of the last pentamer. The inset of b) shows the completion times of the pentamers for the parameter sets analyzed in a) and b), respectively. **c)** Parameter space analysis of hierarchical assembly. The relative yield of full capsids (left) and of clusters of size *n*=55 (right) are depicted using a heat-map representation for various combinations of *k*_*a*_and *k*_*d*_. All data are obtained from 40 independent simulation runs.

Hierarchical assembly of a T1 virus capsid is analyzed in a similar manner as direct assembly. Figures 3a and b show the evolution of relative cluster size population and the average cluster size for assembly under favorable and unfavorable conditions, respectively. Due to the imposed hierarchy, clusters above pentamer size adopt only particular size values (multiples of five). Hierarchical assembly under favorable conditions (Figure 3a) shows a long early phase during which the first pentamers are formed. The following intermediate phase is characterized by addition of newly formed capsomers to one dominant cluster. A striking feature of hierarchical assembly is the dramatic slow-down in the final phase. A majority of trajectories remains in the *n*=55 state for a long time where all but one pentamer have formed and joined the almost complete capsid. This can be explained by increased monomer starvation. In hierarchical assembly, all small clusters of sizes below five are connected by single bonds only. Since the low monomer concentration in the final phase reduces the frequency of diffusional encounter, it takes a long time before the last pentameric ring can be closed irreversibly. From the inset of Figure 3a we clearly identify the formation of the last pentamer as the bottleneck of capsid completion in hierarchical assembly. Here the capsid completion time is plotted against the formation time of the last pentamer for several successful trajectories of one exemplary parameter set. We observe that the completion of the last pentamer is almost instantly followed by its integration into the capsid.

The assembly dynamics shown in Figure 3b for an unfavorable parameter combination does not lead to complete assembly within the given simulation time. In contrast to the favorable case (Figure 3a), the association rate is lower and the dissociation rate is higher, which results in a reduced overall bond stability. This is found to strongly hinder the formation of the late pentamers. Although slower, the overall course of the assembly process is not substantially different from the successful case in Figure 3a. The main difference between the two parameter combinations becomes clear by looking at the completion times of the pentamers which are shown in the inset of Figure 3b. We see that the pentamer FPTs of both cases follow the same shape during the early and intermediate phases, but that for low bond stability the completion times in the late phase are delayed. This delay grows with ongoing assembly, so that the final pentamer does not close within the simulation time. Here the negative effect of low monomer concentration on capsomer assembly, which was discussed earlier, is amplified by the low bond stability.

To quantify the effects of different combinations of *k*_*a*_and *k*_*d*_ on hierarchical assembly of T1, we again perform a systematic investigation of the bond parameter space as shown in Figure 3c. Considering the relative yield of complete capsids (Figure 3c, left image), we observe that only a narrow range of parameters leads to a considerable fraction of successful trajectories. High yield is only observed at high bond stabilities (high *k*_*a*_, low *k*_*d*_) in the upper left corner of the parameter plot. To take into account the critical role of the formation of the last pentamer in our simulations, we also show the yield of almost finished capsids (*n*=55) at the end of the simulation time (Figure 3c, right image). The region where we observe almost finished capsid is considerably expanded and a large fraction of trajectories reaches *n*=55 in the upper left corner of parameter space. The yield decreases along the diagonal from high towards low bond stability values (lower right corner). It becomes clear that the unfavorable parameter combinations do not show kinetically trapped states as they occur in direct assembly, and that most trajectories are close to capsid completion. The high yield of almost finished capsids and the lack of trapped trajectories suggests that the bond hierarchy promotes successful capsid completion, but is vulnerable to monomer starvation.

### T1 direct versus hierarchical assembly

**Figure 4:**
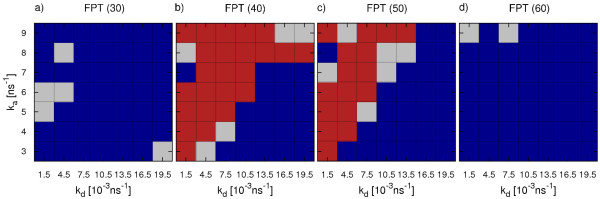
**Comparison of T1 direct and hierarchical assembly.** We evaluate **a)** FPT(30), **b)** FPT(40), **c)** FPT(50) and **d)** FPT(60) for different parameter combinations (*k*_*a*_, *k*_*d*_). Blue fields indicate points at which the respective FPT for direct assembly is smallest while red fields identify hierarchical assembly to be faster. Points where no clear distinction is possible are colored in gray. Every data point is obtained from 45 simulation runs.

The above analysis has revealed a marked difference between direct and hierarchical assembly schemes. From Figures 2 and 3, it is also clear that the final state of a trajectory is strongly affected by the finite length of the simulation and provides only limited information on the dynamics of assembly. In particular hierarchical assembly depends strongly on the formation of the last pentamer and suffers from monomer starvation in the final phase. To evaluate in more detail the performance of the assembly process in its different phases, we systematically compare the FPTs of certain intermediates for both direct and hierarchical assembly. The results are depicted in Figure 4 in a sequence of phase diagrams. Blue areas are those where direct assembly performs better while red indicates parameter combinations where hierarchical assembly is faster. Points where a clear distinction is not possible are shown in gray (difference of direct and hierarchical FPTs less than 10% of the sum of both FPTs).

For the first emergence of intermediates of half the capsid size (FPT(30), Figure 4a), direct assembly is faster throughout the whole parameter space. This is related to the earlier observation of an extended initial phase of hierarchical assembly when compared to direct assembly (see Figure 3). It can be explained by the fact that the monomers in direct assembly exhibit three active binding sites and thus easily form clusters of considerable size. Since hierarchically assembling monomers are designed to form flat pentamer rings, only the two patches forming intra-capsomer bonds are active until full capsomers are formed. Thus the number of fruitful encounters is reduced remarkably, which leads to the observed slow-down of the initial phase.

Looking at the FPTs for the two-third assembled capsid (FPT(40), Figure 4b), we see a large region in the upper left part of the parameter space (high *k*_*a*_, low *k*_*d*_) where hierarchical assembly is now able to overtake direct assembly. This can be attributed to two effects. Firstly, hierarchical assembly speeds up once a pool of capsomers is available. Secondly, direct assembly is slowed down at high bond stabilities. Since the combination of fast formation of large, stable clusters in the early phase (due to high *k*_*a*_) and slow dissociation of small clusters leads to a small number of free monomers, the dominant cluster grows only slowly. In the region of lower bond stability, direct assembly remains faster. Here the increased ability of un- and rebinding of single proteins allows for fast rearrangement, leading to a sufficiently large supply of free monomers so that the dominant cluster can easily grow beyond *n*=40. Simultaneously the pentamer rings in the hierarchical setup form slower than at high bond stabilities.

The difference between the two assembly mechanisms becomes even more evident when looking at FPT(50) (Figure 4c). At low *k*_*d*_values, direct assembly experiences kinetic trapping. As a consequence, hierarchical assembly is superior for almost all small *k*_*d*_values, also at points where the question of dominance remained undecided for FPT(40). The parameter region of weak bonds where direct assembly is faster than hierarchical one is observed to extend during the step from FPT(40) to FPT(50) (lower right corner of parameter space). Under these conditions the effect of beginning monomer starvation delays the pentamer completion of hierarchical assembly.

For the assembly speed of the complete virus capsid (FPT(60), Figure 4d), direct assembly dominates again across almost the whole parameter space. Only at very high bond stabilities hierarchical assembly shows lower or comparable FPT values. This is not surprising taking into account the results for the overall yield of hierarchical assembly (Figure 3c) and underlines the large impact of monomer starvation on hierarchical assembly.

We conclude that hierarchical assembly is not always better than direct assembly. Direct assembly performs better both in the initial and final phases. During the intermediate phase, however, hierarchical assembly is more successful, because it does not suffer from stable bonds preventing structural rearrangements. Due to the limited number of possible interactions, hierarchical assembly is unlikely to get trapped in sub-pentameric units. In general, for hierarchical assembly parameter combinations resulting in high bond stability are favorable. At these values we observe kinetic trapping of most of the directly assembling systems. In addition, the symmetry of the pentamers themselves and the low complexity of their interactions prevent them from getting trapped in large clusters incompatible with the final capsid. For T1, this favors the step-wise build-up of the target structure.

### T1 effect of initial number of monomers

**Figure 5:**
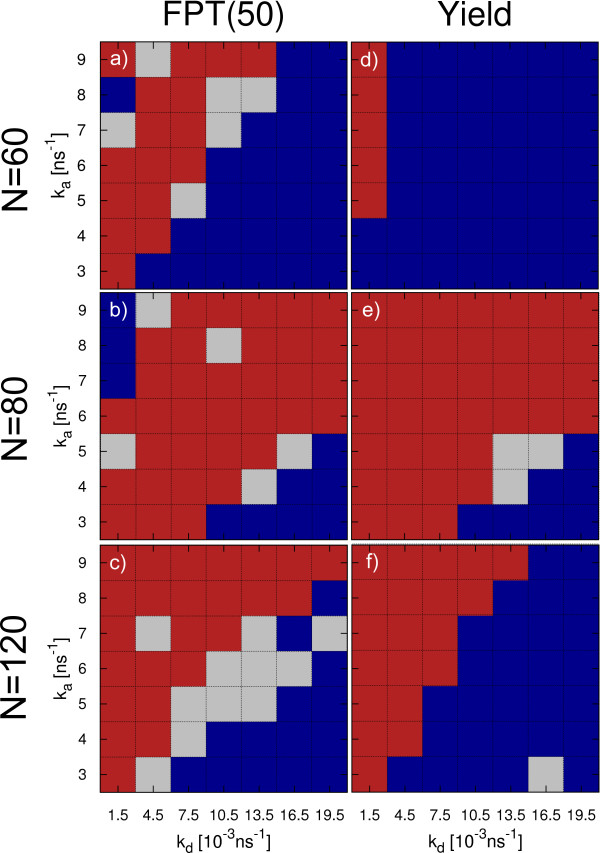
**Effect of initial number of monomers on T1 assembly.** Comparison of FPT(50) **(a-c)** and yield **(d-f)** for direct and hierarchical assembly with an initial number of N=60, N=80 and N=120 monomers, respectively. Blue fields indicate points at which the FPT for direct assembly is smallest or the yield is largest while red fields identify hierarchical assembly to be faster or the yield to be higher. Points where no clear distinction was possible are colored in gray. Every data point is obtained from 45 simulation runs.

Until now we have used exactly as many monomers as needed to form one complete capsid. In experiments, monomers are likely to be present in surplus or to be provided with a certain rate. To study the effect of the limited number of monomers on our simulation, we next increase the initial supply to N=80 and N=120 monomers while keeping the concentration constant by enlarging the simulation box. For the case N=80, a surplus of 20 monomers will be present upon formation of a complete capsid. For the case N=120, two capsids might be formed in parallel and thus the benefit of an increased initial monomer concentration might be shared by them in a complex manner. Figures 5a-5c show a comparison between direct and hierarchical assembly of the FPT(50) for an initial number of N=60, N=80 and N=120 monomers, respectively. As in Figure 4, blue fields indicate that direct assembly has a lower FPT(50), red fields mark parameter pairs for which hierarchical assembly is faster and for gray fields no clear distinction is possible. We see that the comparison of both mechanisms leads to similar results for all setups. When increasing the number of initial monomers we observe a slightly larger region of the parameter space in which hierarchical assembly becomes favorable. This is not surprising, as we identified monomer starvation to strongly hinder the final capsid completion for hierarchical assembly. However, in general the effect of monomer starvation seems to have relatively little impact on the relative efficiency of the two different assembly schemes for clusters of size N=50.

Figures 5d-5f show the yield of the first capsid within simulation time for an initial number of N=60, N=80 and N=120 monomers, respectively. Here again red indicates a higher yield of hierarchical assembly while blue indicates a higher yield of direct assembly. Gray marks parameter pairs with the same yield. In contrast to the FPT(50), we can clearly see that increasing the initial number of monomers results in a largely expanded parameter space in which hierarchical assembly is favorable. This shows that monomer starvation affects the final phase of hierarchical assembly in particular as it has been inferred in the previous section. In fact hierarchical assembly performs well throughout the whole parameter space and shows high yield for intermediate and weak bonds. At very high bond strength we even observe some trapping for hierarchical assembly. However, direct assembly still strongly suffers from kinetic trapping so that the parameter space corresponding to high bond strength remains clearly dominated by hierarchical assembly. We also note that increasing the initial number of monomers from N=80 to N=120 does not lead to a further promotion of hierarchical assembly, presumably because now two capsids form in parallel, each drawing monomers in a similar manner as before for N=60.

To conclude, we find that our main results from the previous section remain valid for an increased number of initial monomers. Hierarchical assembly is favorable at high bond strength due to the decreased possibility of trapping while direct assembly is favorable at low bond strength allowing for fast reorganization of large clusters. In general, we expect that our results also carry over to even larger systems.

### T3 direct versus hierarchical assembly

**Figure 6:**
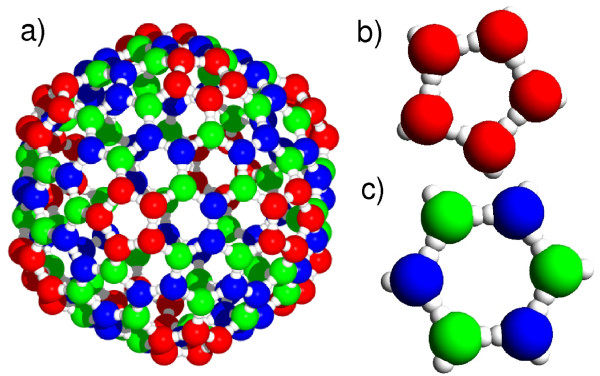
**Model for T3 virus capsid. (a)** Visualization of the T3 virus capsid and its capsomers of **(b)** pentameric and **(c)** hexameric structure. The hexamer is composed of two different protein types.

Given the results for T1 virus assembly, we now ask how they carry over to more complicated geometries like T3 viruses. In this section we compare the characteristics of direct and hierarchical assembly of T3 viruses which are composed of *n*_*f*_=180 monomers. Now we place again exactly the number of monomers needed for the formation of one complete capsid into the simulation box. While in the hierarchical assembly of T1 viruses the capsid was built from pentameric subunits only, T3 virus capsids consist of 12 pentameric and 20 hexameric capsomers. Figure 6 shows a model capsid which, in the hierarchical case, assembles from two different subunits. While the pentamers are formed from identical proteins, the hexamers contain two different particle types. Due to the increased complexity of the T3 capsid, we observe only a small range of bond parameters to lead to high yield for direct assembly in our computer simulations. Moreover, we are not able to identify a parameter combination that allows successful hierarchical assembly within the used simulation time. This is caused by the lowered concentration of individual species of monomers which leads to a dramatic slow-down of capsomer formation in the final phase. As hierarchical assembly reaches the largest cluster sizes at a high association rate of *k*_*a*_=9.0 ns^−1^, we now systematically analyze the effect of different dissociation rates *k*_*d*_(5·10^−4^ ns^−1^≤*k*_*d*_≤1.35·10^−2^ ns^−1^) while keeping *k*_*a*_fixed.

**Figure 7:**
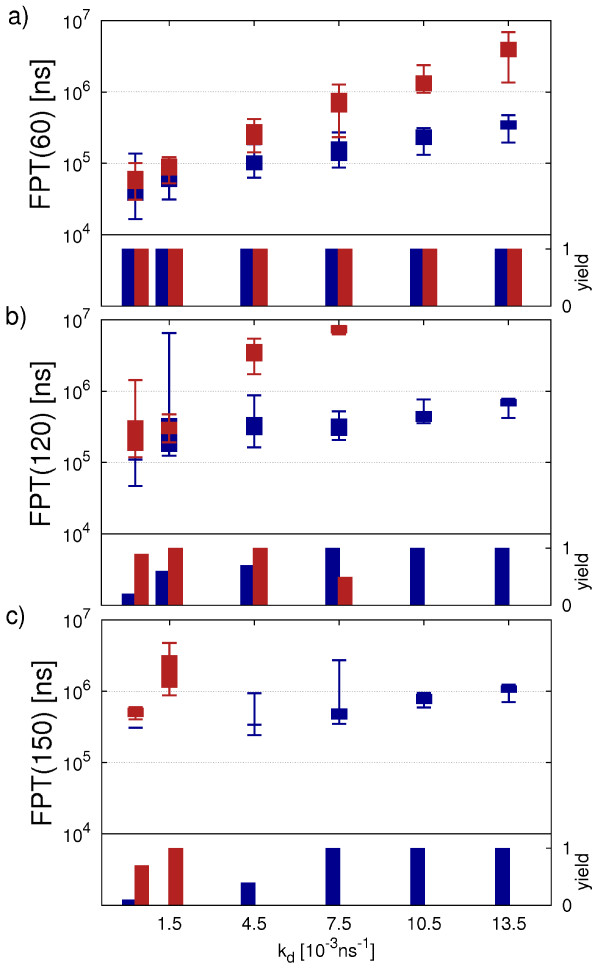
**Comparison of T3 direct and hierarchical assembly. a)**, **b)** and **c)** show the first passage times FPT(60), FPT(120) and FPT(150) together with the relative yield of the corresponding cluster size for fixed *k*_*a*_=9.0 ns^−1^. Blue and red boxes show the results for direct and hierarchical assembly, respectively. The data points are obtained from 10 simulation runs. The maximum simulation length of 9·10^6^ns represents the upper boundary of the FPT values.

Figure 7 shows different FPTs (1/3*n*_*f*_, 2/3*n*_*f*_ and 5/6*n*_*f*_) for direct (blue color) and hierarchical (red) assembly for *k*_*a*_=9.0 ns^−1^and varying dissociation rate. The FPTs are complemented with yield histograms showing the relative number of trajectories which reached the corresponding size within the simulation time. From Figure 7a we immediately see that all trajectories in the investigated parameter interval have grown beyond a cluster size of *n*=60 at the end of the simulation. Comparison of the FPTs for direct and hierarchical assembly reveals assembly speeds of the same magnitude at low values of *k*_*d*_. With growing dissociation rate the FPTs increase for direct as well as for hierarchical assembly. This is not surprising since a lower bond stability leads to an increased number of dissociation events and a slower cluster growth. The FPTs of direct assembly increase only moderately (about one order of magnitude) compared to those of hierarchical assembly (two orders of magnitude). This extreme sensitivity of hierarchical assembly is caused by the strong impact of the low bond stability on capsomers formation. The effect was already observed in hierarchical assembly of T1 and is amplified here due to the presence of several protein types and the resulting lowered effective initial concentration: The number of fruitful monomer encounters is not only reduced by the smaller number of active patches compared to direct assembly, but also by the limited number of suitable binding partners. As a consequence of the dramatic slow-down of hierarchical assembly with increasing *k*_*d*_, we observe zero yield of intermediates of size *n*=120 above a threshold around *k*_*d*_=7.5·10^−3^ ns^−1^(Figure 7b). On the contrary, we record a decrease in the yield of direct assembly below this *k*_*d*_ value. This can be explained with the occurrence of kinetic trapping which we already encountered in T1 direct assembly. Analysis of the corresponding FPT values of so far successful trajectories reveals that, despite the trapping tendency, the speed of direct assembly is still comparable to that of hierarchical assembly at low *k*_*d*_ values. For even larger cluster sizes (FPT(150), Figure 7c) we see further partitioning of the parameter space. Above a threshold around *k*_*d*_=4.5·10^−3^ ns^−1^, no hierarchical assembly is observed, while below this value, only one directly assembling trajectory reaches this size.

### T3 effect of initial number of monomers

**Figure 8:**
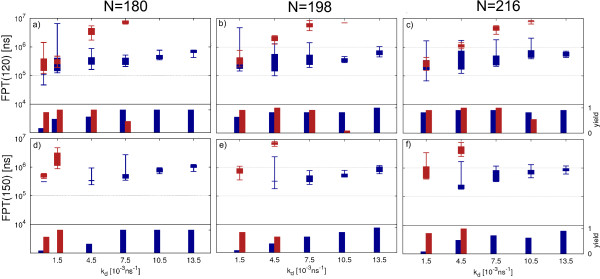
**Effect of initial number of monomers on T3 assembly.** Comparison of T3 direct and hierarchical assembly for an initial number of N=180, N=198 and N=216 monomers. **a)**-**c)** show the FPT(120) and **d)**-**f)** show the FPT(150) together with the relative yield of the corresponding cluster size for the respective initial number of monomers while. Blue and red boxes show the results for direct and hierarchical assembly at a fixed *k*_*a*_=9.0 ns^−1^in a range of *k*_*d*_=1.5·10^−3^ ns^−1^−13.5·10^−3^ ns^−1^. For N=180 a additional value at *k*_*d*_=0.5·10^−3^ ns^−1^is shown. The data points are obtained from at least 10 simulation runs with a maximum length of 9·10^6^*ns*.

As for the assembly of T1 capsids, we again investigate the role of an increased initial number of monomers on the simulation results for the T3 capsid. We increase the initial number of monomers by 10% and 20% (without changing the concentration) and record the FPTs for these simulations. In Figures 8a-8c the FPT(120) and the yield of clusters of size 120 are shown for an initial number of N=180, N=196 and N=216 monomers, respectively. As in the previous section we explore the effect of varying *k*_*d*_ while keeping *k*_*a*_=9.0 ns^−1^fixed. Comparing the FPT(120) for the different setups we see that above *k*_*d*_=1.5 ns^−1^, hierarchical assembly becomes faster for an increased initial number of monomers. Direct assembly in contrast is only slightly affected throughout the parameter space. When looking at the yield of clusters of size 120 within simulation time (9·10^6^ns), we clearly see the positive effect of an increased initial number of monomers on hierarchical assembly for weaker bonds (higher *k*_*d*_). However, it remains worse than direct assembly at these bond strengths. These findings are in agreement with the effect observed for T1 when increasing the initial number of monomers. While the dynamics of direct assembly is only weakly affected by the initial number of monomers, hierarchical assembly suffers less from the effect of monomer starvation. Considering the FPT(150) we again see a complete separation of the parameter space into one region in which only direct assembly is observed and another region in which hierarchical assembly dominates. Looking at the yield we see that for an initial number of 180 monomers hierarchical assembly is only observed for *k*_*d*_≤1.5·10^−3^ ns^−1^ while this region expands to *k*_*d*_≤4.5·10^−3^ ns^−1^for an increased initial number of monomers. It might be possible that the parameter space in which hierarchical assembly is favorable expands further for a larger increase of the initial number of monomers, similar as it was observed for T1 (Figure 5). However, it seems that the favorable effect of an increased initial number of monomers is weaker for T3 capsids than for T1 capsids due to the more complex geometry. In the following section we will investigate the role of complexity of the T3 capsid for the hierarchical assembly of a T3 capsid.

### Capsomer formation in T3 hierarchical assembly

In order to further investigate the effects that slow down hierarchical assembly, we now analyze the dynamics of hexamer and pentamer formation both with computer simulations and a master equation approach. To compare the FPTs for pentamer and hexamer formation, we scale these values with the number of monomers per capsomer ring. This linear scaling is based on the assumption that the mean time for a net addition of monomers to small ring-forming clusters is independent of the cluster size. This simplification in particular neglects the increased number of decay paths of hexamers compared to pentamers. However, the assumption seems justified for the present case of high bond stabilities (high *k*_*a*_, low *k*_*d*_), at least for the early and intermediate phase of assembly.

**Figure 9:**
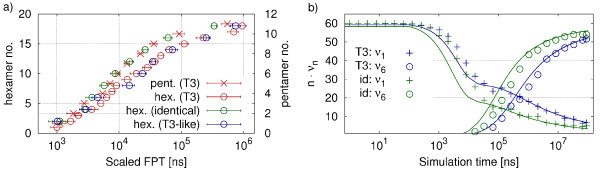
**Analysis of T3 capsomer assembly. a)** FPTs of pentamer and hexamer capsomers emerging during T3 hierarchical assembly are compared to those of T3-like and identical hexamers from the down-scaled simulations. All simulations use *k*_*a*_=9.0 ns^−1^and *k*_*d*_=1.5·10^−3^ ns^−1^. As there are different total numbers of capsomers to be formed of each type, we compare the relative progress of assembly by plotting all species in the same plot with different scales (1 to 12 for pentamers, 1 to 20 for hexamers). FPTs are scaled with the ring size. All values are obtained from 10 independent simulation runs each. **b)** Relative cluster size population *n*·*ν*_*n*_(*t*)during hexamer assembly in the down-scaled systems. The results for monomers (*ν*_1_) and complete capsomers (*ν*_6_) from the analytical master equation approach (lines) are compared to the simulation data (symbols) for T3-like and identical hexamers.

In Figure 9a the average capsomer formation times from T3 assembly at the most promising parameters identified from Figure 7 are shown (now again for N=120). We find that during T3 capsid assembly hexamers form slower than pentamers (for the same sequential number). The difference between the completion times increases with time (the last hexamer data point, no. 18, is an exception to this rule since its FPT is artificially cut down to lower values by the finite length of the simulation). In order to investigate whether this is caused by the different relative densities of monomers forming pentamers (60/180) and hexamers (120/180) or a result of the increased complexity of the hexamer rings, we perform a separate set of simulations. In this complementary simulation we compare the assembly of hexamers consisting of one type of protein (identical hexamers) and hexamers built from two different types of proteins (T3-like hexamers). To reduce the computational effort we downscale the system to half its size while preserving the concentration (i.e. assembly of 10 hexamers in the presence of 30 pentamer-forming monomers). In Figure 9a the hexamer-FPTs from the complementary simulation are compared to those at the same relative positions in the assembly process of the full simulations. The FPTs are again scaled with the ring size. While the dynamics of the identical hexamers follow the course of the pentamers in the full simulation, the FPTs of the T3-like hexamers and the T3 hexamers of the full simulation are in good agreement. This observation suggests that the delay in hexamer formation observed in the full simulation is caused by the two-type complexity of the hexamers compared to the uniformly structured pentamers.

To complement this investigation we use an analytical master equation approach to perform a closer analysis of the dynamics of parallel assembly of several hexamers. Here we develop a set of equations which gives analytic results for the number of clusters of size *n*, *ν*_*n*_ (*t*)(1≤*n*≤*n*_*f*_=6), as a function of association and dissociation rate. The time evolution of the macroscopic quantity *ν*_*n*_is the result of reactions between clusters of all sizes *k* which cause a change of *ν*_*n*_. We introduce the association rate *a* for successful binding of two clusters per unit time and the dissociation rate *b*_*nk*_which denotes the rate for decay of a cluster of size *n* to two daughters of sizes *k* and (*n*−*k*). *b*_*nk*_is composed of the dissociation rate per bond per unit time, *b*, and a factor *d*_*nk*_ which quantifies the probability for the decay of a cluster of size *n* to a constellation where one of the daughters is of size *k*. *d*_*nk*_ is determined by the ratio of total dissociation probability (proportional to the number of bonds which compose *n*) and the probability of the decay products to have the required size. The population *ν*_*i*_increases by the decay of clusters with sizes larger than *i*, so that *d*_*ji*_(*i*<*j*<*n*_*f*_) is always positive. For these cases we find *d*_*ji*_=2 for each pair *j*, *i*, since the decay from 2*i* to two daughters of sizes *i* accounts for a double increase of *ν*_*i*_. The factor *d*_*nn*_denotes the total decay probability of a cluster, it is thus negative and proportional to the cluster size. Here we use *d*_*nn*_=−(*n*−1) for every *n*<*n*_*f*_. We account for one-step processes only, which means we focus on transitions where two clusters merge or one cluster falls apart into two daughter clusters. If we assume that the formation of the complete hexamer ring is irreversible and that the total number of particles *N* is preserved, the complete set of equations describing the time evolution of the system reads

(2) $$\begin{array}{lll} & {\dot{\nu }}_{n}(t)=\underset{\begin{array}{c} \text{growth by association}\\ \text{of smaller clusters} \end{array}}{\underset{︸}{\underset{k+l=n}{\sum }a\nu_{k}(t)\nu_{l}(t)}}-\underset{\begin{array}{c} \text{decrease by association}\\ \text{with other clusters} \end{array}}{\underset{︸}{\nu_{n}(t)\overset{n_{f}-n}{\underset{k=1}{\sum }}a\nu_{k}(t)}}\; & \;\\ & \;\;+\;\;\;\underset{\begin{array}{c} \text{growth/decrease}\\ \text{by dissociation events} \end{array}}{\underset{︸}{\overset{n_{f}-1}{\underset{k=n}{\sum }}b_{\mathrm{kn}}\nu_{k}(t)}}\; \end{array}$$

(3) $$\begin{array}{ll} {\dot{\nu }}_{n_{f}}(t)=\overset{n_{f}/2}{\underset{k=1}{\sum }}a\nu_{k}(t)\nu_{\left(n_{f}-k\right)}(t)\;\;\;\text{(boundary condition)} & \; \end{array}$$

(4) $$\begin{array}{ll} \overset{n_{f}}{\underset{n=1}{\sum }}n\cdot \nu_{n}(t)=N\;\;\;\;\text{(constraint)} & \; \end{array}$$

Numerical evaluation with the initial condition *ν*_1_(*t*=0)=*N*gives the time evolution of all cluster size populations *ν*_*n*_. By fitting the set of equations to the course of all *ν*_*n*_(*t*) from the complementary simulation (*n*_*f*_=6, *N*=60), we obtain parameter combinations *a*, *b* which reproduce the observed assembly dynamics. Under the constraint that the dissociation rate *b* per bond is constant for identical and T3-like hexamers (since the simulations apply the same *k*_*d*_), we find the following parameters: *a*^*id*^=2.4·10^−6^ ns^−1^ (identical hexamers), *a*^*T*3^=1.2·10^−6^ ns^−1^ (T3-like hexamers) and *b*=9·10^−5^ ns^−1^. In general, all *ν*_*n*_(*t*)are reproduced well. This suggests that the assumption of a constant association rate *a* per bond, independent of the sizes of the encountering clusters, is a reasonable approximation for the formation of small rings. The results for *ν*_1_(*t*) and *ν*_6_(*t*) are displayed in Figure 9b together with the simulation data points for both types of hexamers. The early phase is the region which exhibits the largest discrepancies between data and ME results, while the final phase of assembly shows a high level of consistency. This can be explained by the fact that the rate equation framework does not include any spatial constraints and is thus not able to reproduce the same sort of lag time before the first protein reactions as was observed in the simulations, where the randomly distributed particles react only after diffusional mixing leads to the first encounter events. This is also the reason why the difference between the cases of T3-like and identical hexamers becomes visible in the simulation data only after a certain time, while the ME results differ from the very first iteration step (see Figure 9b). Since the rate equations do not contain a diffusional component, the coefficients *a* and *b* cannot be directly related to the simulation parameters *k*_*a*_ and *k*_*d*_. While *k*_*a*_ determines the rate of transition from encounter to a bound state, *a* as well includes the formation of diffusional patch overlap. We estimate *a*=*k*_*a*_/(*N*_*A*_·*V*), where *V * is the simulation box volume. Using the initial concentration *c*=*N*/(*N*_*A*_·*V*), we find the expression $a=k_{a}\cdot \frac{c}{N}$. *a* is thus, as expected, proportional to the initial monomer concentration in the simulation box. Applying this relation to the fit parameters using the effective initial concentration of the protein types, we estimate the overall association rate values to be $\left(k_{a^{\mathrm{id}}}^{\text{fit}}=3.3\cdot 10^{7}\;{\text{s}}^{-1}\;{\text{M}}^{-1}\right)$ and $\left(k_{a^{T3}}^{\text{fit}}=6.5\cdot 10^{7}\;{\text{s}}^{-1}\;{\text{M}}^{-1}\right)$. The fact that the association rate *a*^*id*^ for identical hexamers is about twice the value found for *a*^*T*3^confirms that the difference in assembly dynamics for identical and T3-like hexamers has its origin in a reduced association rate, caused by reduced encounter of matching protein types. Our observations suggest that the association rate decreases linearly with increasing number of bond partners in the system and thus the number of different protein types needed to form a capsomer ring. When comparing our values for the diffusional encounter rate to data from experiments, we see that we overestimate the association rate. In general, the association rate for bimolecular binding reactions is experimentally found to lie between 4·10^6^ and 10^7^ s^−1^ M^−1^[37]. Absence of long-ranged forces, as it is the case for our simulation framework, is predicted to push the rates below 10^6^ s^−1^ M^−1^[42]. The reason for our relatively high estimates for the encounter rate could be the treatment of dissociation as a stochastic event without immediate relocation of the partners. In the present implementation, two patches stay in an encounter after dissociation and their movement is subject to the cluster mobility. We assume this to cause an overestimation of rebinding frequencies which results in an increased association constant. Whereas the association rate constant can be related to other results, there is no such argument for the value of *b*.

## Discussion

Understanding the biophysical principles underlying the self-assembly of virus capsids is of fundamental importance for biology and medicine, and might also promote novel applications in materials science. Here we have presented a Brownian dynamics study of the assembly of icosahedral virus capsids. Using a patchy particle model without potentials, our simulations are relatively fast and therefore we are able to obtain good statistics with relatively modest computing times. One special strength of our approach is the rigorous treatment of translational and rotational diffusion, with the motility matrices for any cluster shape calculated on the fly. Our approach is particularly suited to focus on the effect of a bonding hierarchy on the performance of the assembly process. The hierarchy was established by an event-driven switching of bond characteristics upon the formation of capsomer rings, which have earlier been identified as key intermediate structures of the assembly pathway of some icosahedral viruses [3,26-28,30-32]. We first conducted a detailed comparison of direct versus hierarchical assembly for T1 viruses. To elucidate the effects of an increased complexity of the capsid geometry on the formation of the capsomer rings, we then performed a detailed analysis of capsomer assembly for T3 viruses, including a master equation approach complementing the computer simulations.

Our results for direct assembly of T1 virus capsids show that capsid completion is only successful if the bonds are weak enough to allow for a sufficient number of unbinding and reorganization events. Otherwise kinetically trapped clusters appear. These findings are in good agreement with the results of previous approaches [6,9,40,41]. In marked contrast, hierarchical assembly performs better for high bond stabilities, as the imposed hierarchy reduces kinetic trapping. However, hierarchical assembly is more vulnerable to monomer starvation in the final phase. This effect has previously been observed in other approaches for direct assembly [6,43], but it is even more severe for hierarchical assembly specifically studied here. Comparison of direct and hierarchical assembly reveals that hierarchical assembly, although slower in the early phases, is able to outperform direct assembly at high and intermediate bond strength. This is due to the fact that capsids assembling from highly symmetric capsomers do not require fundamental reorganizations to achieve large cluster size, as it is the case in direct assembly.

The analysis of T3 virus assembly shows that the effects apparent for T1 viruses become amplified by the increased complexity of the capsid geometry. In general, the assembly process of T3 viruses is slower due to the size of the capsid and the increased complexity of the protein interactions. Starting with exactly 180 monomers the parameter space for successful direct assembly is narrowed and we do not observe any complete capsids in hierarchical assembly within the used simulation time. Investigation of the course of assembly of the two mechanisms reveals that they both perform best in distinct regions of the parameter space. Increasing the initial number of monomers we find that hierarchical assembly performs better while direct assembly remains widely unaffected. However, we still observe that both mechanisms are favorable in distinct regions of the parameter space. To analyze the effect of geometric complexity on capsomer formation during hierarchical assembly, we perform a closer analysis of assembly of different capsomer types. The results show a significant slow-down of capsomer formation with increasing structural complexity, which explains why we do not observe any full T3 virus capsids in the hierarchical setup within the given simulation time. These findings suggest a further slow-down for the assembly dynamics of more complex capsids such as T4 and T7 for hierarchical assembly.

Computer simulations of virus assembly are usually carried out with a fixed number of initial monomers and therefore necessarily lead to monomer starvation in the final phase. *In vivo*, this constraint should be less relevant than in our simulations. Once a cell is infected by a virus, one expects to see a constant production rate for viral proteins, and therefore monomer starvation should be less of an issue. It would be interesting to test if in such a situation, hierarchical assembly becomes even more favorable than found here. In computer simulations, this could be done by continuously adding new monomers and removing completed capsids. We leave this to future studies as it would entail to introduce at least two more model parameters, namely the rates for monomer injection and capsid removal, as well as explicit rules on the spatial positioning of the new monomers. A similar study could be done experimentally for viruses which self-assemble *in vitro*, although here too there might be technical problems to implement such procedures. For *in vivo* systems, such studies would depend very much on the details of the virus assembly of interest, in particular on the spatial coordination in regard to the different cellular compartments.

Our simulation framework has great potential for further investigation of assembly of icosahedral viruses, i.e. capsids with higher T-number (T4, T7,...). Although larger simulation times become necessary, they are potentially much smaller than the ones required for less coarse-grained approaches, including patchy particle models with interaction potentials or coarse-grained molecular dynamics simulations. A particular strength of our approach is the possibility to switch patch reactivity during the assembly process. This suggests to investigate even more complex ways to build virus capsids. Our approach could also be applied to other interesting cases of protein assembly, for example to the actin cytoskeleton, for which different regulatory proteins lead to changes in local reactivity.

## Conclusions

We conclude that it might be beneficial for icosahedral viruses to assemble hierarchically, since it prevents kinetic trapping and allows for faster formation of larger structures. Our results suggest that hierarchical assembly performs better than direct assembly for high and intermediate bond stability, while direct assembly is favorable for weak bonds allowing for fast reorganization. For complex viruses, our study suggests that the problem of monomer starvation and critical concentrations has to be addressed for each type of monomer separately, thus making the process more vulnerable for fluctuations in the supply chain and imposing limits to the overall degree of complexity. The partitioning of parameter space into favorable regions for direct versus hierarchical schemes becomes even stronger for more complex capsid geometries and suggests ways to design optimal assembly schemes for different molecular species.

## Competing interests

The authors declare that they have no competing interests.

## Authors’ contributions

JEB and HCRK performed the computer simulations. JEB, HCRK and USS analyzed the data and wrote the paper. USS supervised the project. All authors read and approved the final manuscript.

## Supplementary Material

Additional file 1 Movie showing direct assembly. Representative movie showing the direct assembly of a T1 capsid from 60 monomers. As no switching in bond reactivity occurs, all particles are shown as dark blue spheres with white reactive patches. Snapshots are shown in Figure 1 (top). Click here for file

Additional file 2 Movie showing hierarchical assembly. Representative movie showing the hierarchical assembly of a T1 capsid from 60 monomers. Here the color switch from light blue to red for the intermediate clusters indicates the transition in reactivity upon completed formation of a pentamer ring. Snapshots are shown in Figure 1 (bottom). Click here for file
